# Probiotic Properties of Escherichia coli Nissle in Human Intestinal Organoids

**DOI:** 10.1128/mBio.01470-20

**Published:** 2020-07-07

**Authors:** Suman Pradhan, Alison Ann Weiss

**Affiliations:** a Department of Molecular Genetics, University of Cincinnati, Cincinnati, Ohio, USA; University of California, Irvine

**Keywords:** EHEC, Shiga toxin, bacteriophages, organoid, probiotics

## Abstract

Escherichia coli strain Nissle has been used as a probiotic and therapeutic agent for over a century. Reports suggest that Nissle protects mice from enterohemorrhagic E. coli (EHEC) O157:H7 strains; however, mice are not very susceptible to O157:H7 and are not accurate models for O157:H7 infection in humans. Also, Nissle is closely related to uropathogenic E. coli (UPEC) strain CFT073, suggesting that Nissle could have pathogenic potential. To assess the safety of and protection conferred by Nissle, we modeled infection in stem cell-derived human intestinal organoids (HIOs). HIOs replicate the structure and function of human intestinal tissue. HIOs have a lumen enclosed by a single cell layer of differentiated epithelium, which is surrounded by a diffuse mesenchymal layer. An epithelial barrier which excludes the luminal contents from the surrounding cell layers and medium develops. Nissle appeared to be nonpathogenic; 10^3^ CFU were microinjected into the lumen, and after 3 days, 10^7^ CFU were recovered and the epithelial barrier remained intact. In contrast, microinjected EHEC and UPEC bacteria destroyed the epithelial barrier. To assess the protection conferred by Nissle, HIOs microinjected with Nissle were challenged after 18 to 24 h with EHEC or UPEC. Preincubation with Nissle prevented the loss of the epithelial barrier function, the loss of E-cadherin expression, the increased production of reactive oxygen species, and apoptosis. Nissle did not replicate in the HIO coculture, while the pathogenic strains did replicate, suggesting that Nissle conferred protection via activation of host defenses and not by eliminating competing strains. Nissle was shown to be susceptible to some Shiga toxin phage, and Nissle lysogens could produce Shiga toxin.

## Abstract (precis)

Probiotic, or beneficial, bacteria, such as E. coli Nissle, hold promise for the treatment of human disease. More study is needed to fully realize the potential of probiotics. Safety and efficacy studies are critically important; however, mice are poor models for many human intestinal diseases. We used stem cell-derived human intestinal organoid tissues to evaluate the safety of Nissle and its ability to protect from pathogenic E. coli bacteria. Nissle was found to be safe. Human intestinal tissues were not harmed by the Nissle bacteria introduced into the digestive tract. In contrast, pathogenic E. coli bacteria destroyed the intestinal tissues, and importantly, Nissle conferred protection from the pathogenic E. coli bacteria. Nissle did not kill the pathogenic E. coli bacteria, and protection likely occurred via the activation of human defenses. Human intestinal tissues provide a powerful way to study complex host-microbe interactions.

## INTRODUCTION

Escherichia coli Nissle was one of the first strains used as a probiotic (reviewed in reference 1). It was isolated in 1917 from a German soldier who remained healthy while his comrades succumbed to infections caused by *Shigella*. Nissle is the active strain in Mutaflor, a licensed pharmaceutical widely available in Europe, Canada, and Australia. Nissle has immunomodulatory effects, including suppression of immune-mediated damage as well as upregulation of beneficial responses, and it can improve the intestinal barrier function (1). Furthermore, it can produce bactericidal compounds that reduce the growth of and toxin production by pathogenic bacteria (1). The safety and efficacy of Nissle have been extensively studied. Numerous double-blind placebo-controlled studies have shown that Mutaflor is effective for the treatment and prevention of gastrointestinal disorders, including ulcerative colitis, chronic constipation, Crohn’s disease, and irritable bowel syndrome. It has been shown to be safe when administered to infants (2, 3), although in animal models, dissemination can occur if there is dysbiosis or the host adaptive immunity is defective (4).

Nissle has been reported to protect mice from enterohemorrhagic E. coli (EHEC), including E. coli O157:H7 (5). This is especially important, since no specific therapy for EHEC infection has been approved and antibiotics are contraindicated due to their potential to increase Shiga toxin production (6, 7). However, E. coli O157:H7 is particularly well adapted to cause human disease, and infected animals, such as mice, may remain asymptomatic or display mild symptoms, unless they are compromised in some way (8). How human disease due to EHEC unfolds is poorly understood, and it is especially difficult to assess the effectiveness of probiotics, such as Nissle, in protecting humans from EHEC. Human tissue models can help fill this gap.

Human intestinal organoids (HIOs) are generated from pluripotent stem cells by directed differentiation (9, 10) and represent the small intestine (9). HIOs form spheres with an internal lumen. Unlike cell lines, HIOs possess many cell types. The single cell layer of differentiated epithelium, containing all of the major cell types in the differentiated epithelium (enterocytes, Paneth cells, goblet cells, and enteroendocrine cells), surrounds the lumen. The epithelium is surrounded by mesenchymal cells, expressing myofibroblast and smooth muscle cell markers (9). HIOs have been shown to support the growth of commensal E. coli bacteria but not pathogenic EHEC bacteria (11). Commensal bacteria do not damage the epithelial barrier and remain confined to the lumen, even while reaching densities of about 10^10^ CFU per ml after several days in culture. In contrast, infection of HIOs with EHEC O157:H7 results in cell death and destruction of the epithelial barrier within hours. Organoid destruction appears to be primarily due to bacterium-mediated disruption of the epithelial barrier and not Shiga toxin. At an hour postinfection, E. coli O157:H7 bacteria are seen to be colocalized with epithelial actin, consistent with locus of enterocyte effacement-mediated adherence, and within 4 h, destruction of the epithelial barrier is apparent; however, Shiga toxin at nanogram levels was not detected until 18 h after infection (11). These results were corroborated in studies with HIOs injected with purified Shiga toxin, in which the loss of the epithelial barrier function was not observed until 24 h after injection of nanogram levels of Shiga toxin (12).

We wanted to use HIOs to determine if Nissle confers protection from EHEC infection. However, the genome sequence of Nissle unexpectedly revealed that it is highly related to the uropathogenic E. coli (UPEC) strain CFT073 (13–15). Nissle and CFT073 share many UPEC fitness factors, including the production of intestinal adhesins, capsule, mechanisms for nutrient acquisition (e.g., iron), autotransporters (*pic*, *sat*, and *vat*), the colibactin toxin, and antimicrobial factors designed to reduce microbial competition (microcins H47 and M). However, Nissle lacks two important chromosomal insertions present in CFT073, pathogenicity islands (PAI) PAI1 and PAI2, which encode the hemolysin toxin and the Pap pilus locus, respectively (14). While UPEC strains are not thought to damage the mature human intestinal tract, they have been associated with necrotizing enterocolitis in preterm infants (16). This study had three goals. We wanted to determine if Nissle was nonpathogenic to HIOs and whether it could confer protection from E. coli O157:H7, and we wanted to compare Nissle to the closely related pathogenic UPEC strain. In single-strain infection studies, the UPEC and EHEC strains damaged HIOs; however, Nissle did not cause damage. In coinfection studies, Nissle protected the HIOs from EHEC and UPEC infection.

## RESULTS

### HIOs support the growth of probiotic strain Nissle but not that of EHEC or UPEC.

Previous studies (11) demonstrated that a commensal strain of E. coli lacking virulence factors (strain ECOR13) could grow to a high density in the HIO lumen without the loss of epithelial barrier function. In contrast, a pathogenic EHEC strain (O157:H7 PT29S) caused a rapid loss of the epithelial barrier function. In this study, we wanted to examine whether precolonization with a probiotic strain, Nissle, could protect from EHEC infection in the human organoid model. Since Nissle is highly related to pathogenic UPEC strain CFT073, we examined the pathogenic potential of both Nissle and CFT073. To assess the recovery of the different strains in competition studies, we engineered a different antibiotic resistance marker into each of these strains (Table 1). For simplicity, we refer to kanamycin-resistant Nissle-K as Nissle, gentamicin-resistant CFT073-G as UPEC, and streptomycin-resistant O157:H7 strain PT29S as EHEC.

**TABLE 1:** Bacterial strains used in this study

Strain	Source or description
Nissle 1917	Obtained from Paul S. Cohen, Department of Cell and Molecular Biology, University of Rhode Island, Kingston, RI, USA
Nissle-K	Nissle 1917 transformed with plasmid pBBR1MCS-2, conferring resistance to kanamycin
Nissle::Stx2PT	Nissle-K lysogenized with Stx2 phage from PT29S
PT29S	O157:H7 clinical isolate from CCHMC, a spontaneous streptomycin-resistant isolate (11)
EDL933	O157:H7 hamburger isolate (36), obtained from A. O’Brien
EDL933-G	EDL933 transformed with plasmid pBBR1MCS-5, conferring resistance to gentamicin
CFT073	Obtained from ATCC (WAM2267 ATCC 700928)
CFT073-G	CFT073 transformed with plasmid PBBR1MCS-5, conferring resistance to gentamicin

**FIG 1:**
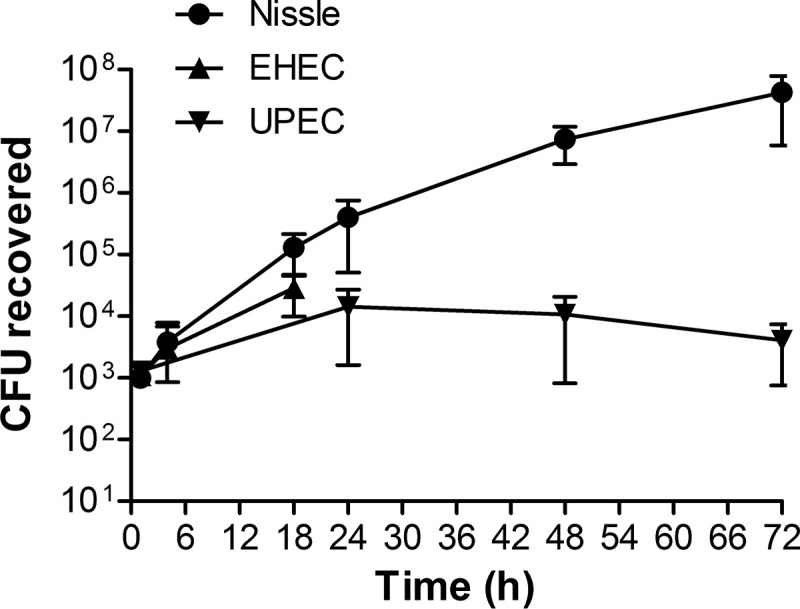
Bacterial growth in HIOs. HIOs were microinjected with 10^3^ CFU of nonpathogenic Nissle, pathogenic EHEC, or pathogenic UPEC. The HIOs were incubated with penicillin-streptomycin in the tissue culture medium to prevent bacterial growth outside of the lumen. The plot shows the mean number of CFU ± SD determined at the indicated times from three different HIOs.

To assess the pathogenic potential of the three strains, approximately 10^3^ CFU were microinjected into the HIO lumen. HIOs were incubated with penicillin-streptomycin in the tissue culture medium to prevent bacterial growth outside of the lumen. After 3 days, >10^7^ CFU per organoid of Nissle were recovered, and as observed previously, EHEC displayed initial growth (Fig. 1A). Previously (11), failure to recover EHEC was shown to be due to a loss of epithelial barrier function, which allowed the antibiotics in the medium to access the bacteria in the lumen. In studies with pathogenic UPEC, viable bacteria were recovered at 72 h postinjection, but they did not grow; only between 10^3^ and 10^4^ CFU were recovered at all time points (Fig. 1A).

### EHEC and UPEC but not Nissle mediate the loss of epithelial barrier function.

**FIG 2:**
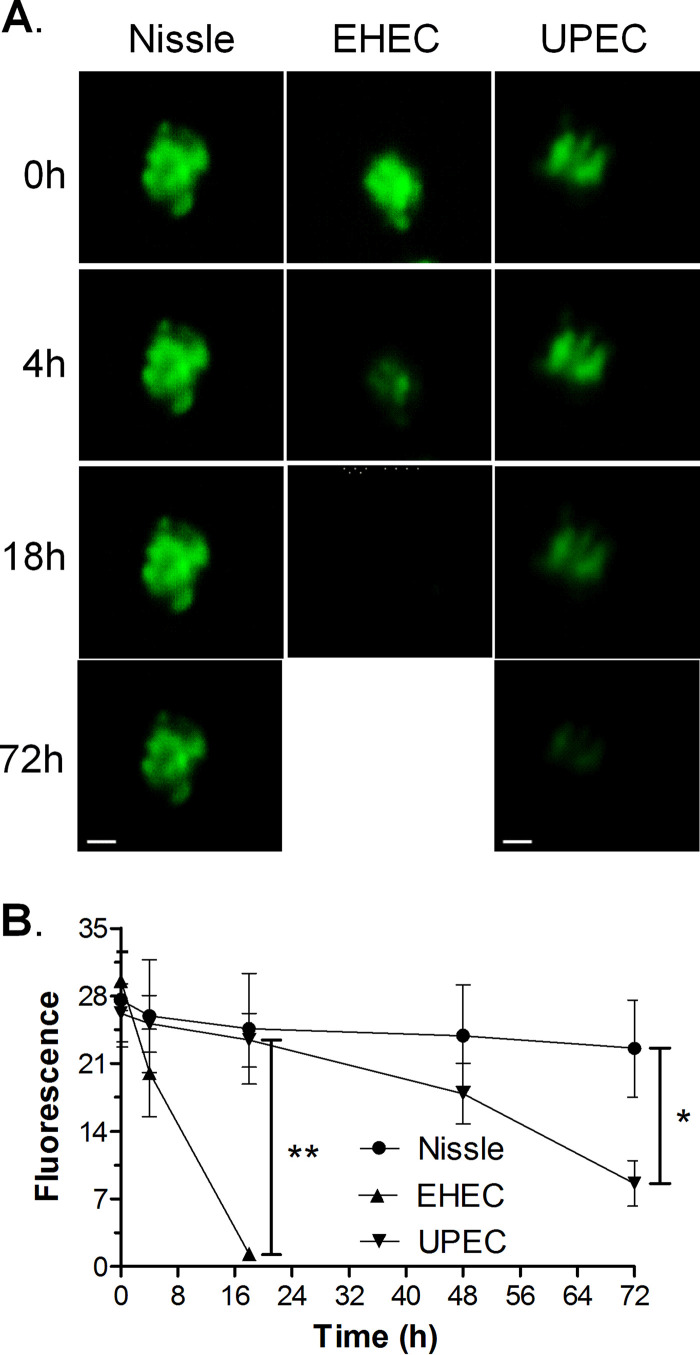
EHEC and UPEC destroy the epithelial barrier. HIOs were injected with 10^3^ CFU of Nissle, UPEC, or EHEC along with the fluorescent dye FITC-dextran. (A) The retention of fluorescence was monitored microscopically over time. Bars, 100 μm. Representative images of experiments performed in triplicate are shown. (B) The epithelial barrier function was assessed by quantifying the retention of fluorescence using ImageJ software, and the fluorescence is plotted as the mean ± SD (*n* = 3). The statistical significance (determined by an unpaired *t* test) of the results for Nissle versus those for EHEC was assessed at 18 h postinfection (**, *P* < 0.003), and the statistical significance of the results for Nissle versus those for UPEC was assessed at 72 h postinfection (*, *P* < 0.02).

To assess whether EHEC and UPEC compromise the epithelial barrier function, HIOs were injected with 10^3^ CFU of Nissle, UPEC, or EHEC along with the fluorescent dye fluorescein isothiocyanate (FITC)-dextran. The epithelial barrier function was assessed by quantifying the retention of fluorescence using ImageJ software (Fig. 2A), and the results were plotted as the mean fluorescence ± standard deviation (SD) (Fig. 2B). Nissle did not cause a loss of epithelial barrier function, since the fluorescence at time zero was not significantly different from the fluorescence at 72 h. Compared to the loss of fluorescence caused by Nissle, EHEC caused a statistically significant loss of fluorescence at 18 h and UPEC caused a statistically significant loss of fluorescence at 72 h.

**FIG 3:**
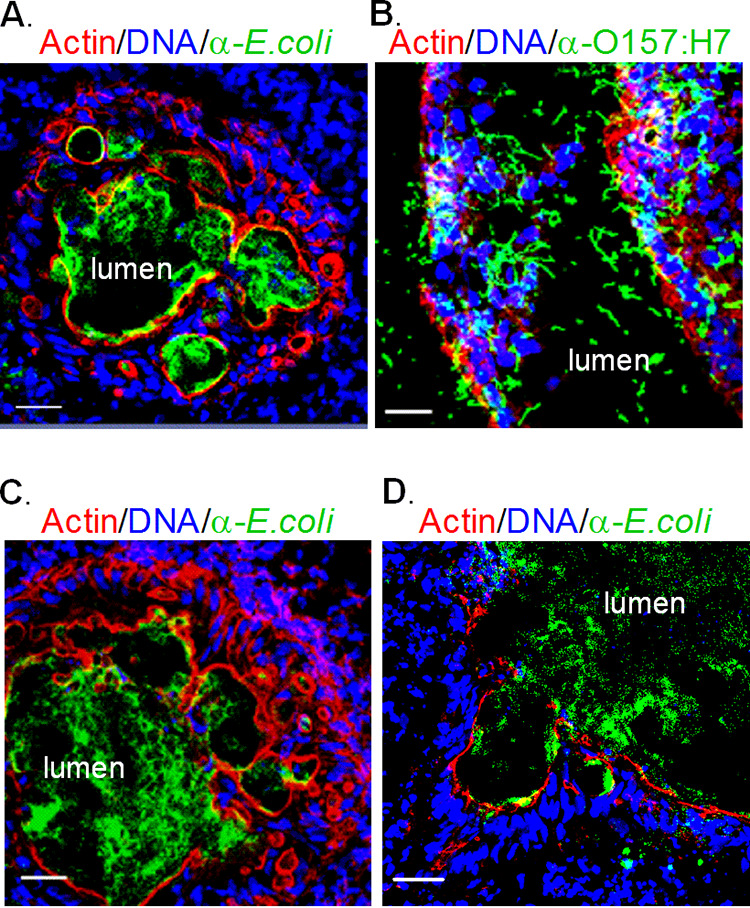
Epithelial damage of infected HIOs. HIOs were microinjected with 10^3^ CFU of Nissle, EHEC, or UPEC and harvested at the indicated times postinjection. Cryosections of infected HIOs were stained for DNA (blue; DAPI), bacteria (green; anti-E. coli for Nissle and UPEC, anti-O157 for EHEC), and F-actin (red; phalloidin) and assessed by confocal microscopy. The lumen is labeled. Bars, 20 μm. Representative images of experiments performed in triplicate are shown. (A) Nissle at 18 hours; (B) EHEC at 18 hours; (C) Nissle at 72 hours; (D) UPEC at 72 hours.

Stained cryosections from the injected HIOs were assessed by confocal microscopy. At 18 h postinjection, Nissle (Fig. 3A, green) was seen in the lumen and was surrounded by a continuous epithelial layer bounded by F actin (Fig. 3A, red). However, in HIOs injected with EHEC, the epithelial layer was damaged, as evidenced by a discontinuous F-actin layer (Fig. 3B, red). Cells were shed into the lumen (Fig. 3B, blue), and rod-shaped bacteria (Fig. 3B, green) had invaded the surrounding epithelial and mesenchymal regions. At 72 h (Fig. 3C), HIOs injected with Nissle were very similar to those at 18 h. However, in HIOs injected with UPEC, the actin layer was discontinuous and the bacteria had invaded the surrounding epithelial and mesenchymal regions (Fig. 3D).

### Nissle protects HIOs from pathogenic EHEC.

**FIG 4:**
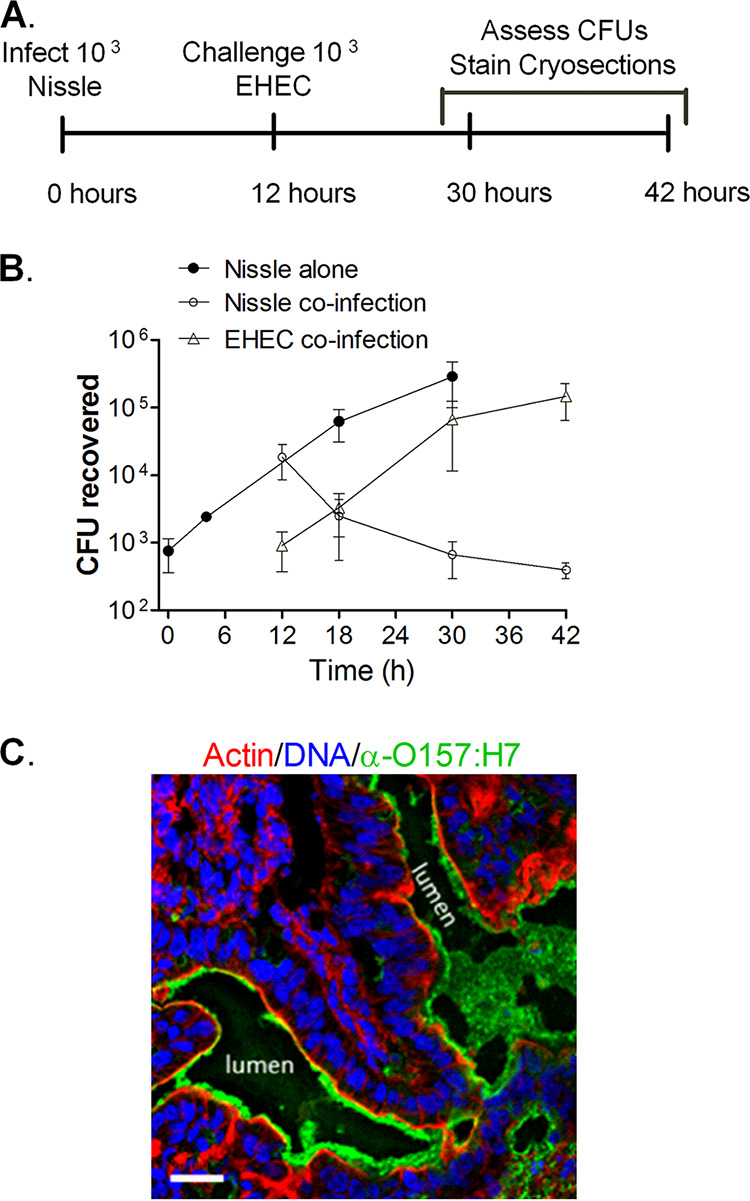
Nissle and EHEC coinfection. (A) Experimental timeline. HIOs were injected with 10^3^ CFU of Nissle. After 12 h, half were challenged with 10^3^ CFU of EHEC. (B) Plot of the mean number of CFU ± SD (*n* = 3) determined at the indicated times. (C) Cryosection of coinfected HIOs at 42 h (30 h after challenge with EHEC) stained for DNA (blue; DAPI), bacteria (green; anti-O157), and F-actin (red; phalloidin). Bar, 20 μm. A representative image of an experiment performed in triplicate is shown.

Nissle has been reported to protect mice from O157:H7 (5, 17). To model superinfection by O157:H7 in an intestine colonized with Nissle (Fig. 4A), approximately 10^3^ CFU of Nissle were microinjected into the HIO lumen and allowed to grow for 12 h. The HIOs were then injected with 10^3^ CFU of EHEC, and the numbers of CFU were determined over time (Fig. 4B). In contrast to HIOs injected with EHEC, from which bacteria were not recovered after 18 h (Fig. 1A), HIOs preinfected with Nissle were able to support the growth of EHEC beyond 18 h, and about 10^5^ CFU were recovered at 42 h, or 30 h postchallenge (Fig. 4B). These results suggest that an intact epithelial barrier prevented access of the antibiotics to the bacteria in the lumen. Interestingly, while EHEC grew in the presence of Nissle, there was a drastic decrease in the number of Nissle CFU, with less than 10^3^ CFU being recovered at 42 h (Fig. 4B, open circles).

Cryosections from the coinfected HIOs were assessed by confocal microscopy at 42 h (Fig. 4C). In contrast to HIOs infected with EHEC alone (Fig. 3B), at 42 h the coinfected HIOs displayed a well-defined, continuous epithelial layer bounded by actin (Fig. 4C, red), with an abundance of EHEC bacteria being seen in the lumen (Fig. 4C, green).

### Nissle confers limited protection from UPEC.

**FIG 5:**
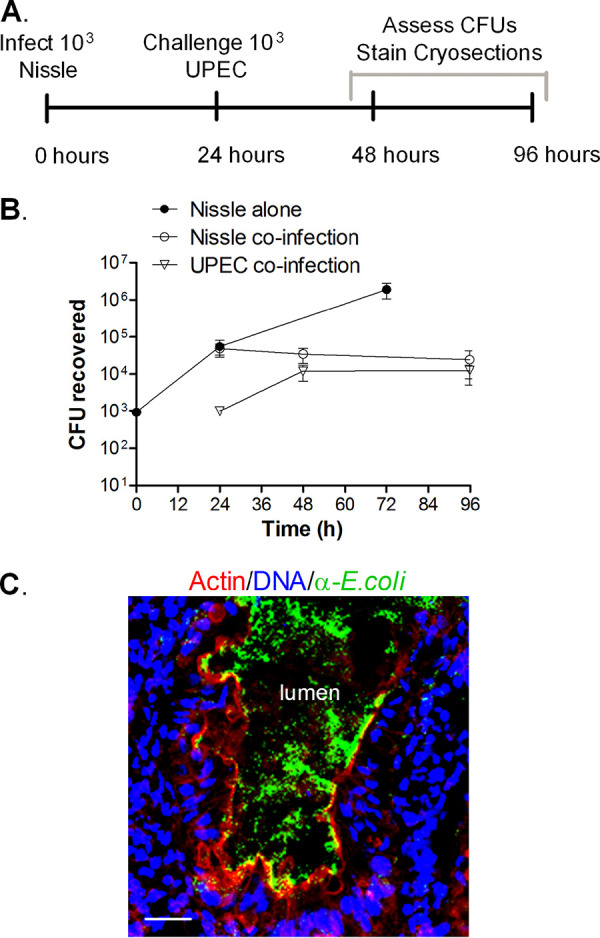
Nissle and UPEC coinfection. (A) Experimental timeline. HIOs were injected with 10^3^ CFU of Nissle, and 24 h later, half were challenged with 10^3^ CFU of UPEC. (B) Plot of the mean number of CFU ± SD (*n* = 3) determined at the indicated times. (C) Cryosection of coinfected HIOs at 96 h (72 h after challenge with UPEC) stained for DNA (blue; DAPI), bacteria (green; anti-E. coli), and F-actin (red; phalloidin). Bar, 20 μm. A representative image of an experiment performed in triplicate is shown.

Superinfection studies were also performed to determine if Nissle conferred protection from UPEC (Fig. 5A). Nissle (10^3^ CFU) was microinjected into the HIO lumen and allowed to grow for 24 h. The HIOs were then challenged with 10^3^ CFU of UPEC, and the numbers of CFU were determined over time. Nissle did not grow after the injection of UPEC (Fig. 5B, open circles). The growth of UPEC was not improved by the presence of Nissle and was very similar to what was seen in pure culture (Fig. 1). The UPEC numbers increased about 10-fold at 24 h after injection and then remained constant (Fig. 5B).

Cryosections from the coinfected HIOs were assessed by confocal microscopy at 96 h (Fig. 5C). The F-actin layer appeared to be continuous (Fig. 5C, red), and the bacteria were mainly confined in the lumen (Fig. 5C, green).

### Nissle protects HIOs from the EHEC- and UPEC-mediated loss of epithelial barrier function.

**FIG 6:**
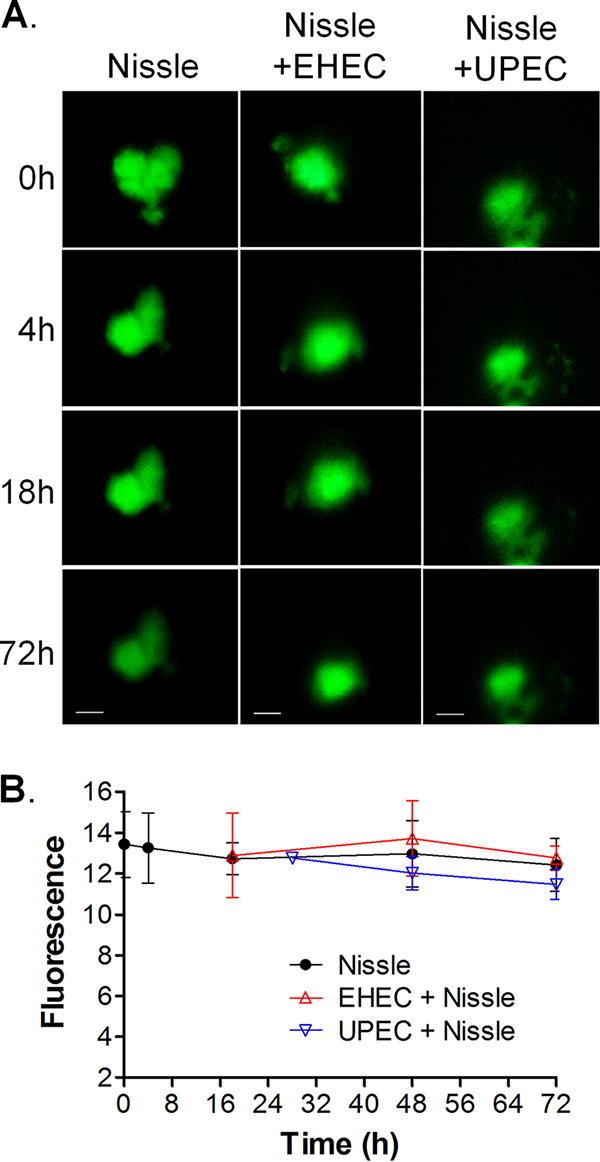
Nissle protects the epithelial barrier function from infection with pathogenic UPEC and EHEC. HIOs were microinjected with 10^3^ CFU of Nissle along with FITC-dextran and challenged with 10^3^ CFU of EHEC at 12 h or UPEC at 24 h. (A) The retention of fluorescence was monitored microscopically over time. Each column represents a single organoid imaged at the indicated time. Bars (displayed only on the final image), 100 μm. Representative images of experiments performed in triplicate are shown. (B) The epithelial barrier function was assessed by quantifying the retention of fluorescence using ImageJ software, and the results are plotted as the mean ± SD, with at least 3 repeats being performed. Differences were not statistically significant at 72 h by analysis of variance with Bonferroni’s multiple-comparison posttest.

Maintenance of the epithelial barrier in coinfection was assessed. Nissle was coinjected with FITC-dextran and challenged with EHEC or UPEC, as described in Fig. 4 and 5. No difference in the loss of fluorescence was seen at 72 h (Fig. 6), suggesting that Nissle protects HIOs against the loss of the epithelial barrier function due to EHEC and UPEC.

### Nissle maintains E-cadherin expression.

**FIG 7:**
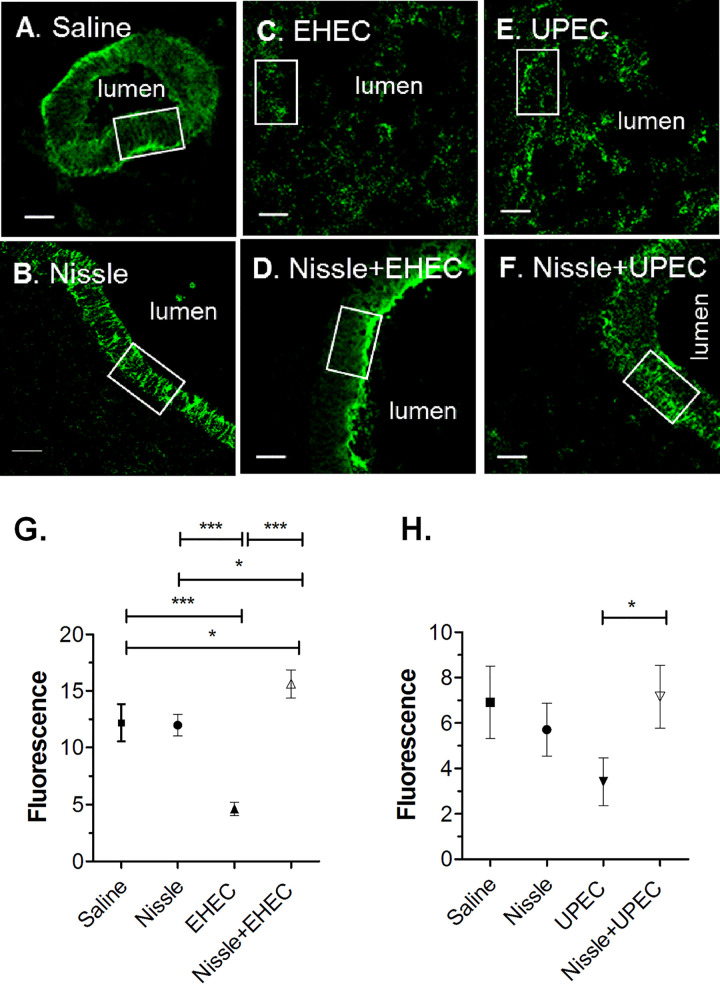
E-cadherin expression. Cryosections from saline-injected controls, single-strain infections, and coinfection experiments (as described in Fig. 4 and 5) were stained for E-cadherin. A white box was sized to outline the epithelial cells stained for E-cadherin in the control HIOs. Keeping the boxed area constant, fluorescence was quantified for three random epithelial areas for each organoid, and the results were averaged. Statistical analysis was performed using the averages for the three independent repeats (*n* = 3). (A) Saline, 72 h postinjection; (B) Nissle, 72 h postinjection; (C) EHEC, 18 h postinjection; (D) Nissle and EHEC, coinfection at 18 h; (E) UPEC, 72 h postinjection; (F) Nissle and UPEC, coinfection at 72 h. White boxes indicate the regions used to quantify fluorescence. Bars, 20 μm. (G and H) Fluorescence was quantified using ImageJ software and plotted as the mean ± SD (*n* = 3). (G) E-cadherin fluorescence in the EHEC challenge; (H) E-cadherin fluorescence in the UPEC challenge. Statistical significance was assessed with GraphPad Prism (version 5) software, using one-way analysis of variance with Bonferroni’s posttest. *, *P* = 0.1 to 0.01; ***, *P* < 0.0001.

E-cadherin is essential for the formation of the adherens junctions that bridge epithelial cells. We assessed E-cadherin expression. Cryosections from HIOs injected with saline or infected as described in Fig. 4 and 5 were stained for E-cadherin (Fig. 7A to F), and fluorescence was quantified (Fig. 7G and H). In HIOs injected with saline or Nissle, a continuous band of E-cadherin was seen surrounding the epithelial cells adjacent to the lumen (Fig. 7A and B, boxes). In HIOs infected only with EHEC or UPEC, E-cadherin was discontinuous and there was no obvious luminal space (Fig. 7C and E). However, in HIOs coinfected with Nissle and EHEC (Fig. 7D) or UPEC (Fig. 7F), the E-cadherin layer was similar to that in HIOs infected with Nissle alone (Fig. 7B). Quantification of E-cadherin fluorescence (Fig. 7G and H) showed a significantly reduced expression of E-cadherin in HIOs infected with EHEC or UPEC alone.

### Nissle protects from EHEC- and UPEC-induced apoptosis.

**FIG 8:**
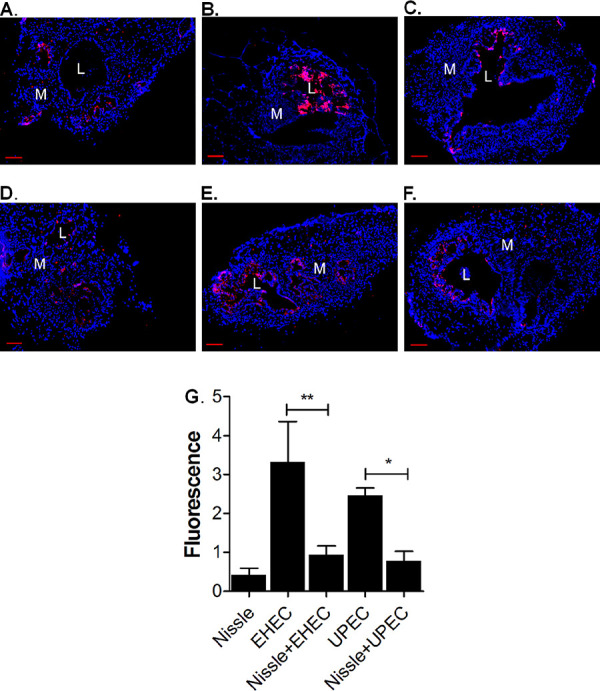
Nissle protects from apoptosis. HIOs were microinjected with 10^3^ CFU of Nissle, EHEC alone, or UPEC alone or coinfected, as described in Fig. 4 and 5. The outer border of the HIO, including the mesenchymal layer, was marked, and the caspase 3 total fluorescence within the area was determined. Values were normalized by dividing the total fluorescence intensity by the area of the organoid. Each infection was performed in triplicate. (A to F) Cryosections of infected HIOs were stained for DNA (blue) and the apoptotic marker activated caspase 3 (red). (A) Nissle, 18 h postinfection; (B) EHEC, 18 h postinfection; (C) Nissle and EHEC, coinfection at 18 h; (D) Nissle, 72 h postinfection; (E) UPEC, 72 h postinfection; (F) Nissle and UPEC, coinfection at 72 h. Bars, 50 μm. L, lumen; M, mesenchyme. (G) Fluorescence was quantified using ImageJ software and plotted as the mean ± SD (*n* = 3). Statistical significance was assessed with GraphPad Prism (version 5) software, using one-way analysis of variance with Bonferroni’s posttest. The results of comparisons between the pathogen with Nissle and the pathogen without Nissle are indicated. *, *P* = 0.1 to 0.01; **, *P* = 0.001 to 0.01.

HIOs were microinjected with 10^3^ CFU of Nissle, EHEC, or UPEC alone or coinfected, as described in Fig. 4 and 5. Cryosections of HIOs preinfected with Nissle and coinfected with EHEC (18 h) or UPEC (72 h) were stained for DNA and activated caspase 3 (Fig. 8A to F, red). Few red-stained apoptotic cells were seen in HIOs injected with Nissle alone, while HIOs injected with EHEC or UPEC alone showed considerable caspase 3 staining. For EHEC, many apoptotic cells were shed into the luminal region. Cellular death (Fig. 8G) was quantified by determination of the retention of fluorescence using ImageJ software. Pathogenic EHEC and UPEC alone caused statistically significantly more apoptosis than Nissle, but precolonization with Nissle protected from apoptosis in coinfection experiments.

### Pathogenic EHEC and UPEC, but not Nissle, stimulate the production of ROS.

**FIG 9:**
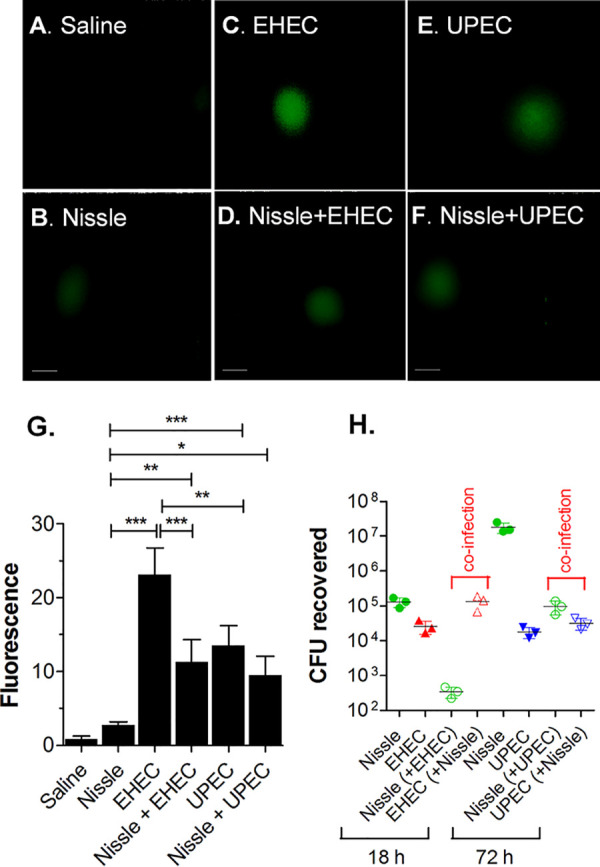
Nissle influences ROS production. HIOs were microinjected with saline; 10^3^ CFU of Nissle, EHEC alone, or UPEC alone; or coinfected, as described in Fig. 4 and 5. (A to F) HIOs were injected with an ROS detection reagent, and ROS fluorescence was measured. (A) Saline, 72 h postinjection; (B) Nissle, 72 h postinjection; (C) EHEC, 18 h postinjection; (D) Nissle and EHEC, coinfection at 18 h; (E) UPEC, 72 h postinjection; (F) Nissle and UPEC, coinfection at 72 h. Bars, 50 μm. (G) Fluorescence intensity was quantified using ImageJ software and plotted as the mean ± SD (*n* = 3). Statistical significance was assessed with GraphPad Prism (version 5) software, using one-way analysis of variance with Bonferroni’s posttest. *, *P* = 0.1 to 0.01; **, *P* = 0.001 to 0.01; ***, *P* < 0.0001. (H) The number of CFU recovered was determined at the indicated time points (*n* = 3).

Pathogenic bacteria can activate innate defenses in the intestine, including the production of reactive oxygen species (ROS), and this was previously demonstrated in HIOs infected with EHEC (11). We wanted to determine if Nissle influenced ROS production. ROS production was assessed by injection of the fluorescent ROS detection reagent at the times indicated below, and fluorescence was assessed microscopically 4 h later (Fig. 9A to F).

The amount of ROS in HIOs infected with Nissle was not statistically different from that in saline-injected organoids (Fig. 9G). However, infection with EHEC and UPEC alone induced significantly more ROS than the amount seen in the saline control and Nissle-infected HIOs. Coinfection with Nissle resulted in a significantly reduced amount of ROS for EHEC but not for UPEC.

### Nissle is susceptible to some but not all Shiga toxin phage.

**FIG 10:**
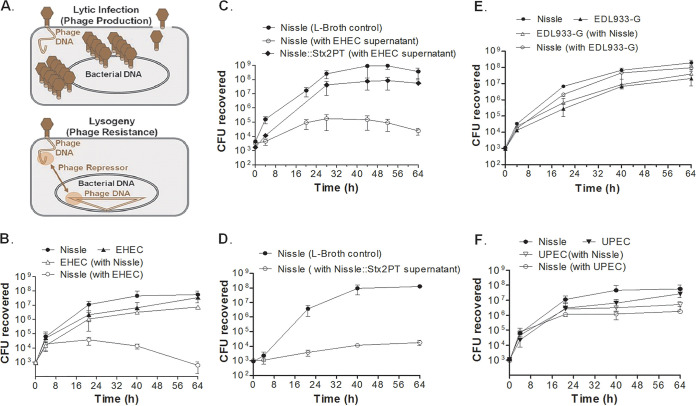
Strain competition in broth culture. (A) Phage life cycle. (Top) Lytic infection results in phage production and bacterial death; (bottom) the repressor produced by lysogenic phage can prevent lytic infection and bacterial death. (B to F) LB broth or filter-sterilized culture supernatant was inoculated with 10^3^ CFU of a single strain or strains mixed 1:1, as indicated, and the numbers of CFU were assessed over time. (B) LB broth was inoculated with Nissle, EHEC, or both. The growth of Nissle was suppressed by EHEC. (C) Nissle was inoculated into LB broth. Nissle and a colony of Nissle that survived coculture with EHEC (Nissle::Stx2PT) were inoculated into the culture supernatant from EHEC. The growth of Nissle was suppressed by the EHEC supernatant. (D) Nissle was inoculated into LB broth or the supernatant from Nissle::Stx2PT. The growth of Nissle was suppressed by the Nissle::Stx2PT supernatant. (E) LB broth was inoculated with Nissle, EDL933, or both. The growth of neither strain was suppressed in mixed culture. (F) LB broth was inoculated with Nissle, UPEC, or both. The growth of neither strain was suppressed in mixed culture.

While Nissle is reported to produce bactericidal molecules, instead of killing EHEC or UPEC, the growth of Nissle was suppressed in HIOs when it was cocultured with EHEC or UPEC. We wanted to determine if Nissle was directly killed by the other E. coli strain or whether the host defenses activated during HIO coculture were involved. In one mechanism known to be used by EHEC (18–20), lysogenic phage occasionally escape repression, enter the lytic cycle, and release infectious phage. Infection of sensitive bacteria by these phage typically results in lytic infection, and the susceptible strain is killed; however, if these phage infect clonally related lysogens that produce the phage repressor, the lytic cycle is aborted and the lysogen is not killed (Fig. 10A). Phage-sensitive strains can become lysogenized, which renders them phage resistant, but it is a rare event. Shiga toxin is phage encoded in EHEC strains (18, 19, 21), while Nissle appears to lack functional phage genomes (22).

Nissle and EHEC were grown in LB broth as pure cultures or mixed 1:1, and the numbers of CFU were determined. Alone, EHEC and Nissle (Fig. 10B, closed symbols) grew to about 10^8^ CFU per ml after 64 h. When mixed 1:1, EHEC (Fig. 10B, open triangles) grew to levels similar to those seen in the absence of Nissle; however, the growth of Nissle was reduced by about 4 log units in coculture (Fig. 1B, open circles).

To determine if survivors of the coculture had become lysogenized, we isolated a colony of Nissle that survived the coculture with EHEC (Nissle::Stx2PT). Since spontaneous phage production releases phage into the supernatant, we tested whether incubation in the EHEC supernatant could kill Nissle and Nissle::Stx2PT. The growth of Nissle incubated in the EHEC supernatant was greatly reduced compared to that of Nissle incubated in LB broth (Fig. 10C). In contrast, Nissle::Stx2PT grew to high levels in the EHEC supernatant. These results suggest that the Nissle survivor of the EHEC coculture had become phage resistant.

The process was repeated using the supernatant from Nissle::Stx2PT. The growth of Nissle was inhibited by coculture in the supernatant from Nissle::Stx2PT compared to the growth of Nissle in LB broth (Fig. 10D). These results suggest that Nissle::Stx2PT was producing phage.

### Nissle lysogens produce Shiga toxin.

**FIG 11:**
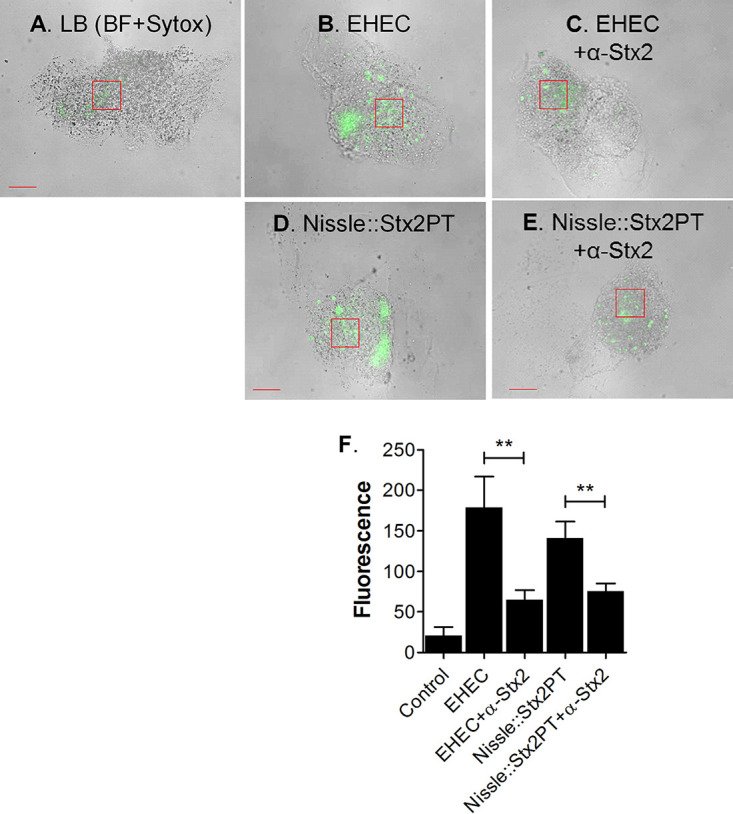
Shiga toxin production by lysogens. HIO mesenchymal cells were incubated for 18 h with LB broth or filter-sterilized supernatant from EHEC or Nissle::Stx2PT in the presence or absence of neutralizing antibodies to the Shiga toxin Stx2a and Stx2b subunits (30 ng each). (A to E) Cellular death was assessed by staining with Sytox green. Representative bright-field (BF) images with fluorescence are shown. Boxed regions represent the areas used to quantitate fluorescence. Bars, 50 μm. (A) LB control; (B) EHEC supernatant; (C) EHEC supernatant and anti-Stx2; (D) Nissle::Stx2PT supernatant; (E) Nissle::Stx2PT supernatant and anti-Stx2. (F) The fluorescence intensity in representative boxed regions was quantified using ImageJ software and plotted as the mean ± SD (*n* = 3). Statistical significance was assessed with GraphPad Prism (version 5) software, using one-way analysis of variance with Bonferroni’s posttest; comparisons between supernatant samples with and without antibody are indicated. **, *P* = 0.001 to 0.01.

Culture supernatant from the presumptive lysogen, Nissle::Stx2PT, was tested for Stx production (Fig. 11). Filter-sterilized culture supernatant was added to isolated HIO mesenchymal cells, previously shown to be susceptible to Shiga toxin (12). In contrast to the findings for the saline control, the supernatant from EHEC and Nissle::Stx2PT caused increased cell death, as evidenced by significantly more staining with Sytox green, a membrane-impermeant DNA dye which can access DNA only when membrane integrity is compromised. Cell death was due to Shiga toxin, as evidenced by rescue when cells were preincubated with neutralizing antibody to Shiga toxin. Together, these results suggest that the failure of Nissle to grow in coculture was not due to factors such as nutrient limitation. Instead, Nissle was susceptible to lytic infection by the Shiga toxin phage. Furthermore, lysogenized survivors became phage resistant and produced Shiga toxin.

### Nissle is resistant to EDL933.

Shiga toxin phage from different O157:H7 strains have different immunity regions, and the resistance or susceptibility of other E. coli strains to the Stx2 phage can vary (21). Previous studies reported that Nissle is able to grow in the presence of the O157:H7 strain EDL933. We examined the susceptibility of Nissle to gentamicin-resistant strain EDL933 (EDL933-G) in coculture. Consistent with these reports, Nissle and EDL933-G grew to similar levels when grown alone or in coculture (Fig. 10E).

### Nissle is not killed by UPEC.

Both Nissle and UPEC strain CFT073 produce microcins, which can kill other bacteria (23). We wanted to determine whether interstrain competition was responsible for Nissle death in HIO cultures. Coculture experiments were performed to determine whether interstrain competition occurs between Nissle and UPEC. When cocultured in LB broth, both strains grew at similar rates and achieved similar final densities compared to those achieved when they were grown alone (Fig. 10F). These results suggest that under these conditions, neither strain impacted the growth of the other.

## DISCUSSION

Assessing the safety and efficacy of Nissle for preventing acute infectious diseases in humans is challenging. While it was once believed that the small intestine was sterile, Zmora et al. used endoscopy to sample the mucosa of the entire human digestive tract (24) and found that all sites were colonized. As expected, the bacterial load in the colonic mucosa was high (10^5^ to 10^7^ CFU). However, colonization was detected in the mucosa of the stomach, duodenum, and jejunum (10^3^ to 10^4^ CFU), and the bacterial load was quite high in samples from the ileum (10^4^ to 10^7^ CFU). It is not clear whether probiotics must be present at the site of infection or whether they can confer protection from a distance. The small intestine is thought to be the site of initial attachment of E. coli O157:H7 (25), and we investigated the probiotic properties of E. coli Nissle in the HIO model system, which represents human small intestinal tissue.

Nissle is highly related to the extraintestinal pathogen CFT073, which was isolated from a patient with pyelonephritis (26). While UPEC strains are not thought to cause intestinal infections in adults, colonization with UPEC strains is associated with the development of necrotizing enterocolitis in preterm infants, and its presence is correlated with death as an outcome (16). HIOs appear to model the intestinal virulence of UPEC. Infection with UPEC strain CFT073 but not with Nissle damaged the HIOs. Nissle and CFT073 share many fitness factors, including adhesins, capsule production, iron acquisition mechanisms, and the production of antimicrobial factors. However, Nissle lacks the CFT073 pathogenicity islands PAI1 and PAI2, which encode the hemolysin toxin and the Pap pilus locus, respectively. Studies are under way to assess the role of the CFT073 pathogenicity islands on virulence in HIOs.

The probiotic Nissle has been reported to employ two general strategies to protect the host from pathogens. The first is related to its ability to confer protection by modifying host defenses (1). As a specific example, the Nissle TcpC protein has been reported to improve the epithelial barrier function by activating the host protein kinase C and extracellular signal-regulated kinase signaling pathways (27). Nissle improved the epithelial barrier function in coinfected HIOs, as evidenced by the increased retention of luminal fluorescence (Fig. 6) and enhancement of E-cadherin expression (Fig. 7). Nissle has also been reported to upregulate the expression of both pro- and anti-inflammatory pathways (1). We observed Nissle to downregulate inflammatory defenses. Precolonization with Nissle greatly reduced ROS production (Fig. 9) and cell death due to apoptosis (Fig. 8). While we do not have any evidence regarding the specific properties of Nissle that may confer protection, epithelial maturation may be partially involved. Within 4 h of infection with a commensal or pathogenic E. coli O157:H7 strain, upregulation of the transcription of proteins that participate in gastrointestinal defenses was seen, including the mucins (MUC2 and MUC13), the structural component of gastric mucus; trefoil factor (11).

The second general strategy involves Nissle’s ability to outcompete other bacterial strains. Nissle is highly effective at intestinal colonization due to its intestinal adherence mechanisms. Nissle has multiple iron acquisition systems (1) and can successfully compete for other nutrients (5, 17). In the mouse intestine, Nissle can use both glycolytic and glyconeogenic nutrients (28). The “restaurant” hypothesis (29) proposes that organisms with the same nutritional preferences cannot coexist unless they occupy spatially distinct regions (i.e., different “restaurants”), where they can obtain nutrients locally. This allows established, commensal E. coli bacteria to outcompete invading E. coli pathogens. However, pathogenic EHEC and UPEC strains also possess adherence mechanisms and iron and nutrient acquisition systems, and those of Nissle do not appear to be superior. The protection mediated by Nissle does not appear to act by outcompeting either EHEC or UPEC strains.

Also related to strain competition, Nissle has mechanisms to kill other bacteria, including genes for the antibacterial compounds microcin H49, microcin M, and colibactin (14). The production of microcin M was shown to limit the growth of Salmonella enterica in the inflamed mouse intestine via a siderophore-dependent process (30). However, in our HIO coinfection studies, the luminal recovery of Nissle declined, making it unlikely that Nissle protects the HIOs by deploying its antimicrobial defenses. This was confirmed in broth coculture. Neither EHEC nor UPEC was killed by coculture with Nissle in LB broth. This was not unexpected for UPEC. Strains which produce antibacterial compounds possess resistance mechanisms to prevent self-destruction. Since the highly related Nissle and UPEC strains share the same antimicrobial defenses, they must possess the same resistance mechanisms.

Phage production by lysogenic strains can be another antibacterial weapon. Nissle has six chromosomal regions that are predicted to encode partial prophage genomes (22). While none of these regions appears to be capable of producing intact phage, one region does encode a phage repressor that confers resistance to bacteriophage lambda-like phage, including phage from O157:H7 EDL933. The introduction of the Nissle phage repressor gene into a phage-sensitive E. coli strain conferred phage resistance. Nissle also appears to have extracellular defenses that inactivate phage (22). Consistent with this report, we found that Nissle is resistant to EDL933-G in coculture. However, another study reported that Nissle could limit the growth of Shiga toxin-producing E. coli strains, including strain EDL933 (31). In this study, quantitative PCR of the Shiga toxin gene was used as an indirect method to determine EDL933 abundance. Genes for Shiga toxin are encoded in the phage genome. During lysogeny, the phage are integrated into the chromosome, and there is a one-to-one correspondence between phage genomes and E. coli chromosomes. However, during lytic replication the phage are excised from the chromosome, and the number of phage genomes compared to the number of chromosomal equivalents is greatly increased. As part of the phage genome, the number of Shiga toxin genes is also greatly amplified, and this method cannot be reliably used to predict bacterial numbers.

In contrast to EDL933-G, Nissle was killed by coculture with another O157:H7 strain, EHEC PT29S, both in HIOs and in broth. Phage are highly recombinogenic, recombination can generate extensive phage mosaicism (32), and Shiga toxin-encoding phage are highly diverse (21). The phage immunity region encodes the phage repressor. The repressor protects from infection by a phage with the same immunity region, but when a phage infects a strain with a different immunity region, repression does not occur. Typically, lytic infection results, but occasionally, the strain may become lysogenized. We have shown that Nissle is killed by the Stx2 phage from PT29S, and Nissle survivors become lysogenized and produce Shiga toxin.

The use of Nissle as a therapeutic intervention for EHEC infection has been proposed (33). While the authors of that study cite the phage resistance of Nissle to be an important safety characteristic (33), they did not examine enough isolates. O157:H7 has complex interactions with commensal bacteria (34), and these depend on the identity of the O157:H7 stain as well as the identity of the commensal strains. The coculture of O157:H7 with phage-susceptible strains can result in increased Shiga toxin production *in vitro* and *in vivo* (18–20). Furthermore, microcin, a bacteriocin also produced by Nissle, has been shown to increase Shiga toxin production by an SOS-dependent pathway (35), similar to the way in which the antibiotic ciprofloxacin induces Shiga toxin production (19). Our demonstration that Nissle is phage susceptible and that lysogens can produce Shiga toxin seriously compromises the use of Nissle as a therapeutic.

The use of probiotics, or beneficial bacteria, to fight infection and promote intestinal health is very attractive. However, many human intestinal pathogens cannot be adequately modeled in cell culture or experimental animals, such as mice, and it is not clear whether information from these surrogate infection models is reliable enough to guide medical practice. Stem cell-derived human tissues, such as HIOs, provide a powerful alternative to traditional experimental approaches.

## MATERIALS AND METHODS

### Bacterial strains.

The bacterial strains used in this study are listed in Table 1. Antibiotic resistance markers were introduced to distinguish the different strains in mixed cultures. Briefly, kanamycin resistance was introduced into Nissle, gentamicin resistance was introduced into CFT073, and streptomycin resistance was introduced into O157:H7 strain PT29.

### Preparation, microinjection, and characterization of HIOs.

HIOs, prepared by directed differentiation of an H1 human embryonic stem cell line, were obtained from the Pluripotent Stem Cell Facility and Organoid Core at Cincinnati Children’s Hospital and Medical Center at the spheroid stage. Spheroids were embedded in Matrigel basement membrane matrix (catalog number 356234; BD Biosciences), to support the development of the 3-dimensional architecture, and maintained in reconstituted gut media (11). Bacterial cultures for HIO challenge were grown as previously described (13). Briefly, the Nissle, EHEC, and UPEC strains were grown in LB broth to an optical density of about 1.0. The cultures were serially diluted in sterile normal saline (catalog number 114-055-721; Quality Biologicals) to 1 × 10^7^ CFU per ml, and 100 nl containing approximately 10^3^ CFU of E. coli was microinjected into the HIO lumen. The HIOs were incubated with penicillin-streptomycin (catalog number 15140-122; Gibco) in the tissue culture medium to prevent bacterial growth outside of the lumen. For coinfection studies, HIOs were microinjected with 10^3^ CFU of Nissle and challenged with 10^3^ CFU of EHEC after 12 h or 10^3^ CFU of UPEC after 24 h. Microinjection of saline was used as a negative control.

### Confocal microscopy.

HIOs were cryosectioned and stained following the protocol previously described (11). The histologic stains used in this study were Texas Red-X phalloidin (catalog number T7471; Molecular Probes), 4′,6-diamidino-2-phenylindole (DAPI; catalog number D3571; Invitrogen), antibody against E. coli (catalog number 1001; Virostat), antibody to E. coli O157:H7 (catalog number ab20976; Abcam), and caspase 3 (catalog number ab13847; Abcam). The epithelial marker E-cadherin (catalog number 1416; Abcam) was used to study the extent of epithelial damage. The fluorescence within representative areas was quantified for three independent samples for each experimental condition.

To study cellular death during apoptosis, HIOs were stained for caspase 3 after the coincubation time points. Fluorescence was quantified for three independent samples for each experimental condition.

### Epithelial barrier.

To assess the maintenance of the epithelial barrier function, bacteria were diluted 1:1 with FITC-dextran (average molecular weight, 3,000 to 5,000; catalog number FD4; Sigma-Aldrich) at a 25 μM concentration, and luminal fluorescence was monitored under a fluorescence microscope (Nikon Eclipse TE2000-U) over time.

### Reactive oxygen species.

To assess ROS production, infected HIOs were injected with 100 nl of 300 pM ROS detection reagent (catalog number ENZ-51011; Enzo Life Sciences) at the times indicated above. The HIOs were further incubated at 37°C with 5% CO_2_ in a humidified chamber for 4 h and imaged using a fluorescence microscope (Leica DMi8 wide-field microscope). Fluorescence was quantitated by use of the image-processing program ImageJ.

### Bactericidal activity in broth.

LB broth (10 ml) was inoculated with 10^3^ CFU of an individual strain or a 1:1 mixture of 10^3^ cells of each strain for coculture experiments, and the bacteria were grown at 37°C (without shaking). The bacteria were plated on L agar with stain-specific antibiotic selection at various times to determine the number of CFU per organoid. Kanamycin was used to identify Nissle, streptomycin was used to identify EHEC PT29, and gentamicin was used to identify UPEC and EDL933. The bactericidal activity in the culture supernatants was assessed using filter-sterilized supernatants from overnight cultures grown in LB broth at 37°C. Briefly, the cultures were centrifuged at 4,696 × *g* for 10 min to pellet the bacteria and sterilized by passage through a 0.2-μm-pore-size filter (catalog number SLGVM33RS; Millipore).

### Shiga toxin assay.

Shiga toxin toxicity for mesenchymal cells was assessed as previously described (12). Briefly, day 28 HIOs were pipetted up and down to separate the mesenchymal cells from the epithelial layer. The isolated mesenchymal cells were embedded in 10 μl Matrigel, with 400 μl of gut medium being added to each well. The cells were incubated for 18 h with 400 μl gut medium and 100 μl LB (negative control), 400 μl gut medium and 100 μl of filter-sterilized EHEC supernatant (positive control), or 400 μl gut medium and 100 μl filter-sterilized supernatant from Nissle::Stx2PT. To determine whether toxicity was due to Shiga toxin, mesenchymal cells were preincubated with neutralizing antibody to the Stx A subunit (catalog number NR845; Biodefense and Emerging Infections Research Resources Repository [BEI]) and Stx B subunit (catalog number NR-9352; BEI) at 30 ng each for an hour before addition of supernatants. Cell death due to necrosis was assessed by addition of Sytox green (0.5 μM) for 30 min, and the cells were imaged under a Leica DMi8 wide-field microscope. Fluorescence within representative areas was quantified for three independent samples for each experimental condition.

### Quantification and statistical analysis.

All experiments were performed with at least 3 independent repeats. The fluorescent signal was quantified using Image J software. Statistical analyses were done using GraphPad Prism (version 5) or Excel software.

### Data availability.

We will make the data fully available without restriction.
